# A Hydrogen-Sulfide-Repressed Methionine Synthase *SlMS1* Acts as a Positive Regulator for Fruit Ripening in Tomato

**DOI:** 10.3390/ijms232012239

**Published:** 2022-10-13

**Authors:** Zhi-Kun Geng, Lin Ma, Yu-Lei Rong, Wan-Jie Li, Gai-Fang Yao, Hua Zhang, Kang-Di Hu

**Affiliations:** 1 School of Food and Biological Engineering, Hefei University of Technology, Hefei 230009, China; 2 Key Laboratory of Cell Proliferation and Regulation Biology, Ministry of Education, College of Life Science, Beijing Normal University, Beijing 100875, China

**Keywords:** *Solanum lycopersicum*, methionine synthetase, fruit ripening and senescence, ethylene, hydrogen sulfide (H_2_S)

## Abstract

Ethylene is a key phytohormone that regulates the ripening of climacteric fruits, and methionine is an indirect precursor of ethylene. However, whether methionine synthase plays a role in fruit ripening in *Solanum lycopersicum* (tomato) is still unknown. In this study, we find that a tomato methionine synthase (named *SlMS1*), which could be repressed at the transcriptional level by hydrogen sulfide (H_2_S), acts as a positive regulator for tomato fruit ripening. By a bioinformatics analysis, it is found that SlMS1 and SlMS2 in tomato are highly homologous to methionine synthases in *Arabidopsis thaliana*. The expression pattern of *SlMS1* and *SlMS2* is analyzed in tomato, and SlMS1 expression is up-regulated during fruit ripening, suggesting its potential role in regulating fruit ripening. A potential bipartite nuclear localization signal is found in the amino acid sequence of SlMS1; thus, SlMS1 is tagged with GFP and observed in the leaves of *Nicotiana benthamiana*. Consistently, SlMS1-GFP shows strong nuclear localization and also cytoplasmic localization. The role of *SlMS1* in regulating fruit ripening is investigated in tomato fruit by transient silencing (virus-induced gene silencing, VIGS) and transient overexpression. The results show that *SlMS1* silencing causes delayed fruit ripening, evidenced by more chlorophyll and less carotenoid accumulation, while *SlMS1* overexpression accelerates fruit ripening significantly compared with control. Further investigation shows that *SlMS1* overexpression could up-regulate the expression of carotenoid-synthesis-related genes (*PSY1*, *PDS*, *ZDS*), chlorophyll-degradation-related genes (*NYC1*, *PAO*, *PPH*, *SGR1*), cell-wall-metabolism-related genes (*CEL2*, *EXP*, *PG*, *TBG4*, *XTH5*) and ethylene-synthesis-pathway-related genes (*ACO1*, *ACO3*, *ACS2*), while *SlMS1* silencing causes the opposite results. The correlation analysis indicates that *SlMS1* expression is negatively correlated with chlorophyll content and positively correlated with carotenoid and ripening-related gene expressions. Taken together, our data suggest that SlMS1 is a positive regulator of tomato fruit ripening and a possible target gene for the ripening-delaying effect of H_2_S.

## 1. Introduction

Tomato fruit is rich in nutrients and thus a popular healthy vegetable. With a clear genetic background and short growth cycle, tomato is a model organism for the research on plant growth and fruit ripening and senescence. Tomato is a typical climacteric fruit, and ethylene plays a key role in tomato fruit ripening [1]. Tomato fruit ripening is a complex and orderly physiological and biochemical process, accompanied by fruit softening, chlorophyll decomposition and carotenoid accumulation. At the level of the subcellular structure, the transition from chloroplast to chromoplast occurs, and the thylakoid structure of chloroplast disintegrates and loses the ability of photosynthesis [2]. Fruit degreening is a visible sign during tomato fruit ripening. The degradation of chlorophylls begins with the conversion of chlorophyll b to chlorophyll a [3]. Subsequently, the chlorophyll degradation pathway could be further catalyzed by the Pheophorbide a monooxygenase (PAO) pathway and the Pheophytin pheophorbide hydrolase (PPH) pathway. In addition, SGR (STAY-GREEN) proteins could promote chlorophyll degradation during tomato fruit ripening [4,5]. During the transition from chloroplast to chromoplast, a large amount of carotenoid accumulates in the chromoplast mediated by phytoene synthase (PSY), phytoene desaturase (PDS), ζ-carotene isomerase (ZISO), ζ-carotene desaturase (ZDS), etc. [6]. Moreover, fruit softening during the ripening process involves a coordinated expression of a large number of genes. Many cell-wall-modifying enzymes cooperate to induce changes in texture, leading to cell wall loosening and hydration of cell wall polymers. The cell-wall-modifying enzymes include pectinesterase (PE), pectate lyase (PL), polygalacturonase (PG) and β-galactosidase (β-GAL), and the hemicellulose/cellulose-modifying enzymes: 1,4-glucanase, xyloglucan transglycosylase/hydrolase (XTH) and expansin (EXP), etc. [6]. Expansins promote cellulose microfibrils to slide over one another, and PG reduces the chain length of pectins [7]. Due to the strong respiration, texture softening and the consumption of fruit nutrients, the shelf life of tomato fruit is shortened during the postharvest storage, resulting in a substantial waste of resources. Therefore, further exploration of the regulation mechanism of tomato fruit ripening and senescence will be helpful for the research on the mechanism of tomato fruit development.

Tomato is a typical climacteric fruit, as it undergoes a rapid rise in respiration and a burst of ethylene production at the onset of the ripening process. The pathway of ethylene biosynthesis involves the conversion of S-adenosylmethionine (SAM) to 1-aminocyclopropane-1-carboxylic acid (ACC) by ACC synthase (ACS) and then by ACC oxidase (ACO) to ethylene [8,9]. In tomato, *ACO1* and *ACO3* expression increases strongly and correlates well with the autocatalytic rise in ethylene production, suggesting their important role in regulating fruit ripening [10].

Methionine is an indirect precursor for ethylene biosynthesis, and we propose that its influx into the ethylene biosynthesis pathway is critical for ethylene burst during fruit ripening. SAM is generated with methionine as the substrate under the catalyzation of S-adenosylmethionine synthase [11]. Given that ethylene is a key phytohormone regulating tomato fruit ripening and senescence, we believe that methionine is a key metabolite controlling the ethylene pathway. During de novo methionine synthesis, methionine synthase in plants converts homocysteine to methionine using N5-methyltetrahydrofolate as a methyl donor [12,13]. In tomato, the functions of methionine synthase are not well studied. Methionine synthase ER69, i.e., *SlMS1*, corresponding to *Solanum lycopersicum* methionine synthase in this work, was found to be an ethylene-regulated gene, suggesting that methionine synthase may regulate tomato fruit ripening [14].

Hydrogen sulfide (H_2_S) can alleviate ripening and senescence of multiple postharvest fruits, such as strawberry, kiwifruit and tomato, by activating the antioxidant system and antagonizing the ethylene pathway [15,16,17]. However, the key point gene for the target of H_2_S in mediating its ripening delay remains unclear. As methionine synthase is the determinant for the influx to the ethylene pathway, we propose that methionine synthase genes may be the targets for H_2_S signal. In this study, we studied the role of *SlMS1* in regulating fruit ripening by transient silencing (virus-induced gene silencing, VIGS) and transient overexpression of *SlMS1* in tomato fruits. We found that *SlMS1* showed both nucleus and cytoplasm localization, and it is a positive regulator for tomato fruit ripening.

## 2. Results

### 2.1. Identification and Bioinformatics Analyses of Methionine Synthase Genes in Tomato Genome

**Figure 1:**
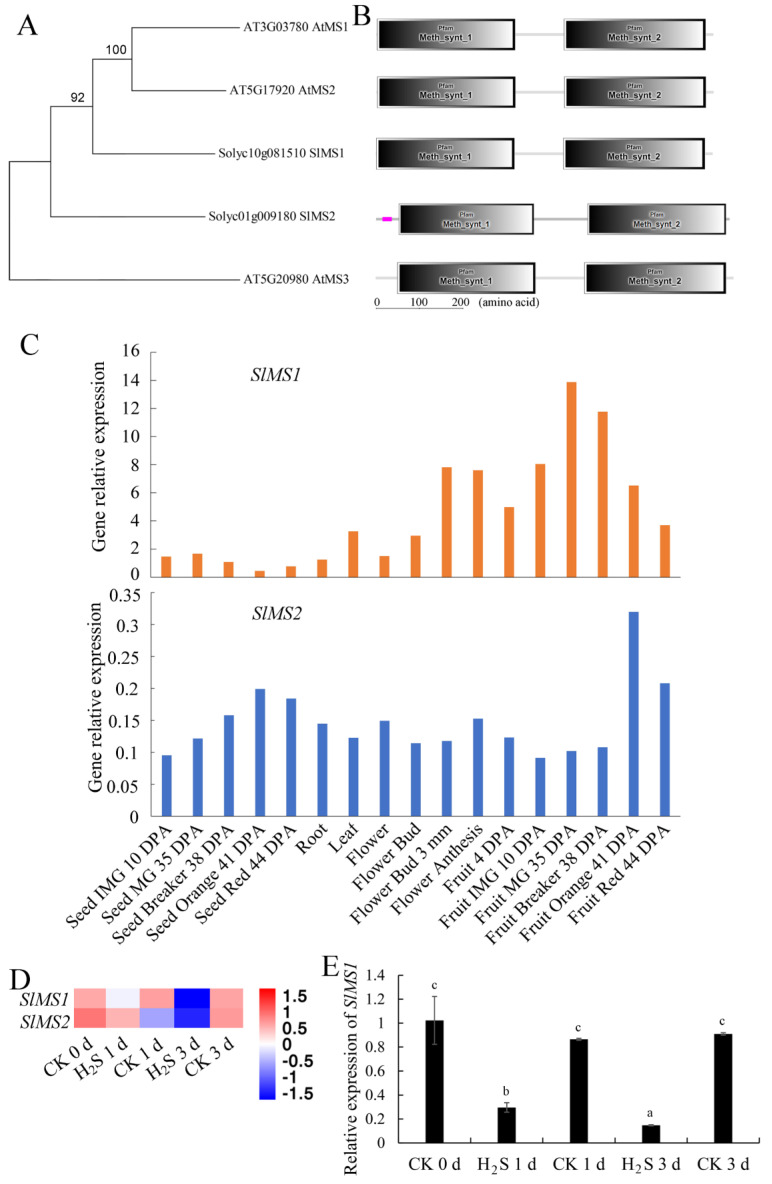
Phylogenetic analysis, domain construction and gene expression analysis for methionine synthases in Arabidopsis and tomato. (**A**) The neighbor-joining method was used to perform a phylogenetic analysis of methionine synthase in Arabidopsis and tomato based on a neighbor-joining method with 1000 bootstrap value. (**B**) Domain constructions of methionine synthases in Arabidopsis and tomato. (**C**) Exploration of transcription of methionine synthase genes in different tissues of tomato by public data. The original expression data were obtained from the online tool http://tomexpress.toulouse.inra.fr/query, accessed on 21 August 2022, and the tomato cultivar was Micro Tom. (**D**) Heatmap of *SlMS1* and *SlMS1* transcriptional level in tomato fruit treated with H_2_S (H_2_S) or the control (CK) for 0, 1 and 3 days. (**E**) Relative expression of *SlMS1* in tomato fruit treated with H_2_S (H_2_S) or the control (CK) for 0, 1 and 3 days. Different letters above the bar stand for significant difference between two samples (*p* < 0.05, as determined by Student’s *t*-test, respectively).

The amino acid sequence of AtMS1 (AT3G03780) obtained from NCBI was used as a query to obtain homologous proteins in *A. thaliana* and *S. lycopersicum.* Five methionine synthase proteins were identified and, as shown in the phylogenetic tree in Figure 1A, the identified methionine synthases could be classified into two groups, in which AtMS3 forms a single branch, and the other proteins, AtMS1, AtMS2, SlMS1 and SlMS2, group into a single branch. Moreover, we analyzed the functional domain and found that all methionine synthases in Arabidopsis and tomato show two conserved domains, i.e., Meth_synt_1 and Meth_synt_2. Meth_synt_1 could be found in Cobalamin-independent methionine synthase, MetE, which catalyzes the synthesis of the amino acid methionine by the transfer of a methyl group from methyltetrahydrofolate to homocysteine. The Meth_synt_2 domain is referred to as the N-terminal half and C-terminal half of MetE in *E. coli*, which show some sequence similarity, indicating that the *MetE* gene has evolved from an ancestral *MetE* gene by duplication. Therefore, sequence alignment was performed, as shown in Figure S1, and it can be found that methionine synthases are highly conserved in *A. thaliana* and *S. lycopersicum.* Additionally, the tertiary structures of methionine synthases in *A. thaliana* and *S. lycopersicum* are predicted and shown in Figure S2. The dimensional structures are very similar among the proteins, and a cleft can be found between the two domains. The cleft may be the site for the reaction of 5-methyltetrahydrofolate + L-homocysteine to generate tetrahydrofolate + L-methionine.

The analysis of the physicochemical properties of AtMS1 showed that AtMS1, AtMS2 and SlMS1 showed the same 765 amino acids and similar pI values, which were 6.09, 6.09 and 6.01, respectively. Consistently, AtMS1, AtMS2 and SlMS1 with similar physicochemical characteristics show more intimate phylogenetic relations.

### 2.2. Expression Patterns of Tomato MS Gene in Different Tissues of Tomato Plants and in Response to Hydrogen Sulfide Treatment

To explore the expression patterns of *MS* genes in tomato plant, the transcription data were explored in tomato roots, stems, leaves, flower buds, flowers and fruits at different ripening stages in a public database. As shown in Figure 1C, there were significant differences in the expression of *MS* genes in different tissues of tomato. *SlMS1* showed an increasing trend of expression until the mature green stage, followed by decline, suggesting that *SlMS1* might play an important role in regulating tomato fruit ripening. H_2_S could act as a repressive signal for fruit ripening by antagonizing the effect of ethylene in tomatoes. Thus, we attempt to establish whether H_2_S could repress the expression of *SlMS1* and *SlMS2* in tomato fruit. We explore previous published transcriptome data, where mature green tomato fruits were treated with H_2_S donor NaHS harvested at 0, 1 and 3 days after storage [18]. As shown in Figure 1D, H_2_S application repressed the expression of *SlMS1* and *SlMS2* at 1 and 3 days of treatment and the original transcriptome data were presented in Table S1. As shown in the q-PCR result of Figure 1E, the expression of *SlMS1* in H_2_S-treated fruit was less than half of the control at 1 and 3 days of treatment, suggesting that the expression of *SlMS1* was repressed by H_2_S.

### 2.3. Subcellular Localization of SlMS1

**Figure 2:**
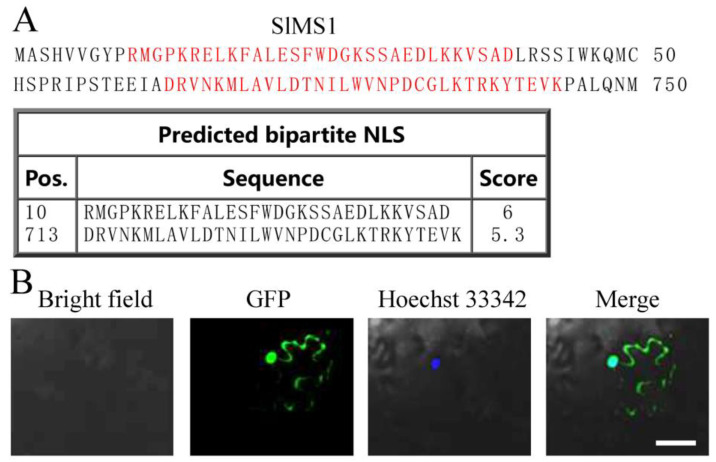
Nuclear localization signal prediction and subcellular localization of *SlMS1* in tobacco cells. (**A**) Nuclear localization signal of *SlMS1* was predicted on https://nls-mapper.iab.keio.ac.jp/cgi-bin/NLS_Mapper_form.cgi, accessed on 12 August 2022, and the red letters stand for potential nuclear localization signal. (**B**) Confocal image of tobacco leaf cells expressing *SlMS1*-GFP. Scale bar: 50 µm.

Subcellular localizations of methionine synthases in *A. thaliana* and *S. lycopersicum* were predicted by an online tool, and the results are shown in Table 1. It can be found that AtMS1 shows cyto: 7, chlo: 4, mito: 2, nucl: 1, AtMS2 cyto: 5, mito: 4, chlo: 3, nucl: 1, pero: 1 AtMS3 mito: 9, chlo: 5, SlMS1 cyto: 7, chlo: 3, mito: 2, nucl: 1, pero: 1, SlMS2 mito: 9, chlo: 5. As *SlMS1* might play a role in regulating fruit ripening, the potential nuclear localization signal in SlMS1 was further predicted by NLS Mapper with a cut-off score of 5. As shown in Figure 2A, two potential bipartite NLS sites were found, where one score was 6, and the other was 5.3. Basic amino acids, such as K or R, are important amino acids that define NLS. The first NLS sites contained KR and KK separated by a linker composed of 19 amino acids, and the NLS localized in the N-terminal region of SlMS1, all suggesting that the first NLS is a real bipartite NLS. Then, the recombinant *SlMS1*-GFP plasmids were expressed in tobacco leaves, and fluorescence was observed by a co-focal microscope. As shown in Figure 2B, *SlMS1*-GFP co-localized with the nucleus stain Hoechst 33342, indicating that the localization of SlMS1 was mainly in the nucleus. Moreover, *SlMS1*-GFP also showed cytoplasm localization.

### 2.4. Effects of Transient Silencing and Gene Expression of SlMS1 on Tomato Fruit Ripening

**Figure 3:**
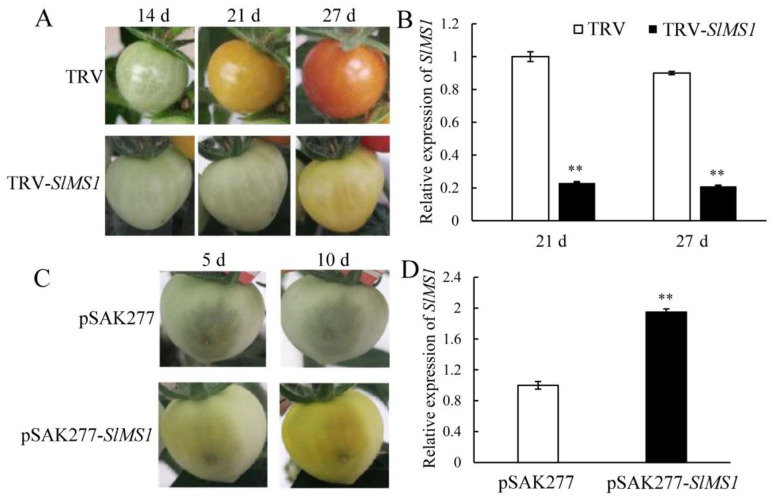
Transient silencing and transient overexpression of *SlMS1* in the ripening process of tomato. (**A**) Phenotype of *SlMS1*-silenced fruit in tomato. TRV: co-inoculation of the empty vectors TRV1 and TRV2 as control; TRV-*SlMS1*: co-inoculation of TRV1 and TRV2-*SlMS1*. (**B**) RT-qPCR of *SlMS1* in control (TRV) and *SlMS1*-silenced fruit (TRV-*SlMS1*) at 21 and 27 days post inoculation. (**C**) Transient overexpression of *SlMS1* by injection of pSAK277 (control) and pSAK277-*SlMS1* photographed at 5 and 10 days post injection. (**D**) Relative gene expression of *SlMS1* in control (pSAK277) and *SlMS1*-overexpressed (pSAK277-*SlMS1*) fruit at 10 days post inoculation. The symbol ** stands for and *p* < 0.01, as determined by Student’s *t*-test.

To investigate the role of *SlMS1* in regulating tomato fruit ripening, the expression of *SlMS1* was transiently silenced by VIGS, and gene expression was achieved by the transient expression of pSAK277-*SlMS1* in tomato fruit. The recombinant plasmids TRV-*SlMS1* and pSAK277-*SlMS1* were transformed into Agrobacterium to infect tomato fruits at 15 days after anthesis and mature green fruits, respectively. As shown in Figure 3A, the phenotype of *SlMS1*-silenced fruit in tomato was observed at 14, 21 and 27 days post infection (DPI). At 21 DPI, the color of the fruits of the control group began to change to yellow, but no color change occurred with TRV-*SlMS1*. At 27 DPI, the fruits of the control group almost turned red, while TRV-*SlMS1* were still in the breaker color stage. To confirm the successful silencing of *SlMS1* by VIGS, the expression of *SlMS1* was examined by RT-qPCR (Figure 3B), and it could be found that *SlMS1* was greatly silenced by VIGS compared with the control, suggesting the efficiency of gene silencing. Thus, the above results indicated that the silencing of *SlMS1* delayed tomato fruit ripening, implying that *SlMS1* is a positive regulator of fruit ripening.

Additionally, the tomato fruit chamber was injected with Agrobacterium harboring pSAK277-*SlMS1*, which increases the expression of *SlMS1* by the 35S promoter. Figure 3C showed that the fruit of *SlMS1* pSAK277-*SlMS1* showed earlier fruit ripening than the control group in the injection chamber at 5 and 10 DPI, indicating that *SlMS1* may play a role in promoting fruit ripening. The efficiency of gene overexpression of pSAK277-*SlMS1* was analyzed by RT-qPCR. The data in Figure 3D indicate that the expression of *SlMS1* was increased significantly in pSAK277-*SlMS1* infected fruit. Therefore, *SlMS1* is a positive regulator of tomato fruit ripening.

### 2.5. Effect of Gene Silencing and Overexpression of SlMS1 on the Metabolism of Chlorophyll and Carotenoid in Tomato Fruit

**Figure 4:**
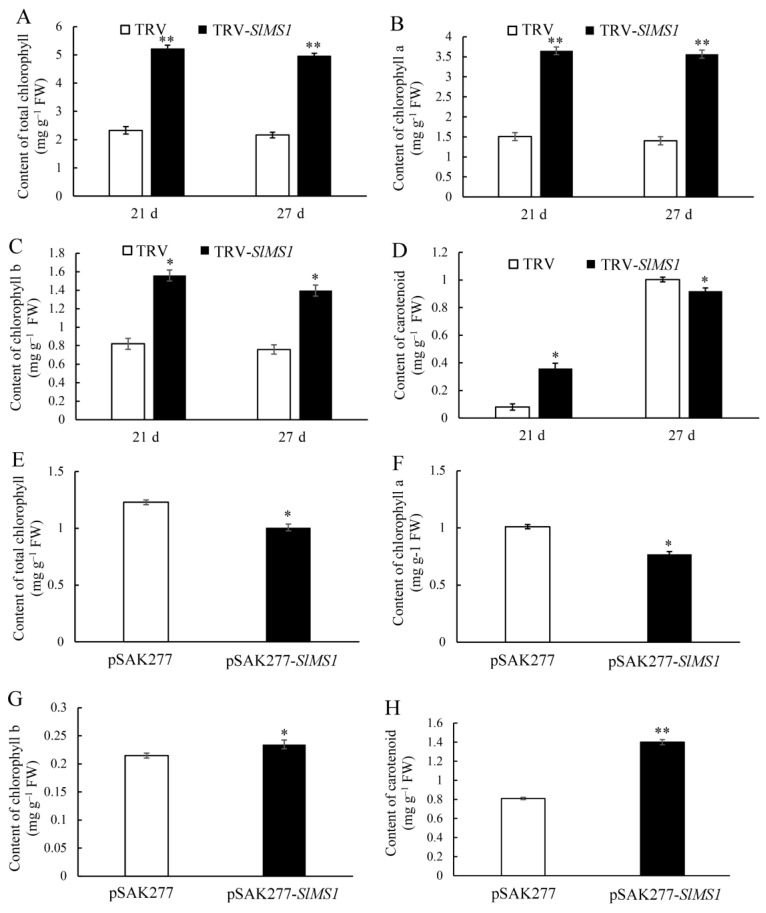
Effect of transient silencing and transient overexpression of *SlMS1* on pigment content in tomato fruit. (**A**–**D**) Content of total chlorophyll (**A**), chlorophyll a (**B**), chlorophyll b (**C**), carotenoids (**D**) in control (TRV) and *SlMS1*-silenced fruit (TRV-*SlMS1*) at 21 and 27 days post inoculation. (**E**–**H**) Content of total chlorophyll (**E**), chlorophyll a (**F**), chlorophyll b (**G**), carotenoids (**H**) in control (pSAK277) and *SlMS1*-overexpressed (pSAK277-*SlMS1*) fruit at 10 days post inoculation. The symbols * and ** stand for *p* < 0.05 and *p* < 0.01, as determined by Student’s *t*-test, respectively.

The degradation of chlorophyll and the accumulation of carotenoids are important characteristics of the ripening process of tomato fruit. In order to further study the mechanism of *SlMS1* gene silencing and transient expression in affecting tomato fruit ripening, the carotenoid and chlorophyll contents were determined in control fruit, TRV-*SlMS1* and pSAK277-*SlMS1* infected fruit. As shown in Figure 4A, the content of total chlorophyll decreased slightly in both control and TRV-*SlMS1* at 21 and 27 DPI during fruit ripening, whereas the content in *SlMS1*-silenced fruit was about two-fold of that in the control. The data of chlorophyll a and chlorophyll b showed a similar trend to total chlorophyll content (Figure 4B,C). The carotenoid content in *SlMS1*-silenced fruit at 21 DPI was higher than that of the control group, but at 27 DPI, the content in the control was slightly higher than that of the *SlMS1*-silenced fruit (Figure 4D). The carotenoid content in pSAK277-*SlMS1* infected fruit at 27 DPI was significantly higher than that of the control group, while the chlorophyll in the fruit of the pSAK277-*SlMS1* plant was much lower than that of the control group (Figure 4E–H). The above results indicated that *SlMS1* silencing decreased the accumulation of carotenoid, while *SlMS1* overexpression improved the biosynthesis of carotenoid.

### 2.6. Effect of Gene Silencing and Overexpression of SlMS1 on the Expression of Carotenoid Synthesis Genes and Chlorophyll Degradation Genes

**Figure 5:**
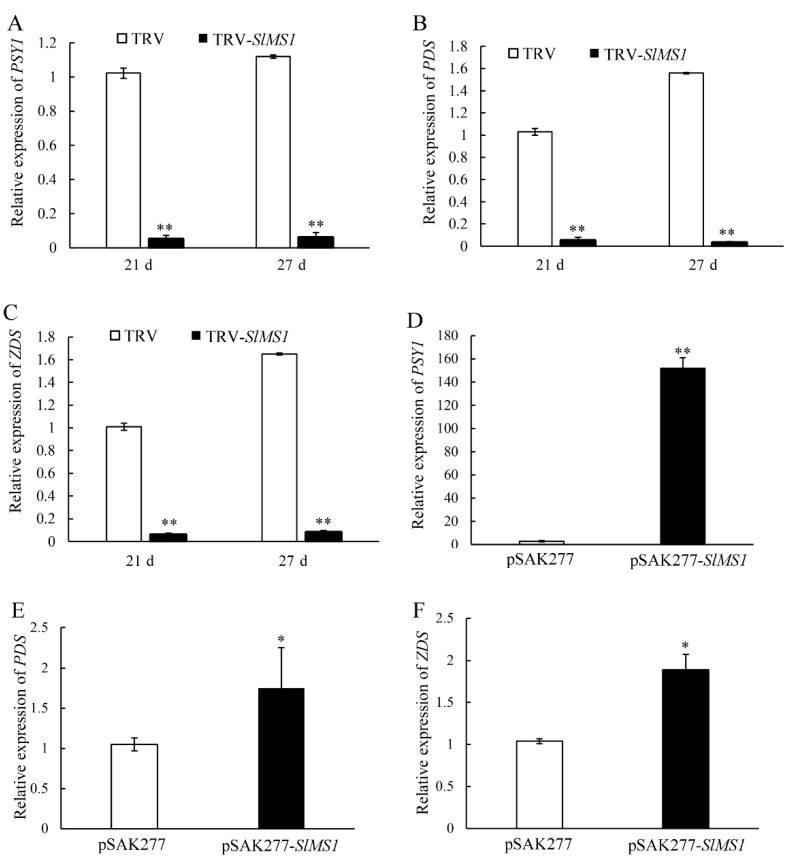
Effect of transient silencing and transient overexpression of *SlMS1* on gene expression of lycopene biosynthesis related genes *PSY1, PDS* and *ZDS* in tomato fruit. (**A**–**C**) Relative gene expression of *PSY1* (**A**), *PDS* (**B**) and *ZDS* (**C**) in control (TRV) and *SlMS1*-silenced fruit (TRV-*SlMS1*) at 21 and 27 days post inoculation. (D-F) Relative gene expression of *PSY1* (**D**), *PDS* (**E**) and *ZDS* (**F**) in control (pSAK277) and *SlMS1*-overexpressed (pSAK277-*SlMS1*) fruit at 10 days post inoculation. The symbols * and ** stand for *p* < 0.05 and *p* < 0.01, as determined by Student’s *t*-test, respectively.

To explore the mechanism of differences in carotenoid metabolism in *SlMS1* silenced and overexpressed fruit, the expression levels of carotenoid-synthesis-related genes *PSY1*, *PDS* and *ZDS* were analyzed by RT-qPCR. As shown in Figure 5A–C, the expression of *PSY1*, *PDS* and *ZDS* in control increased gradually from 21 to 27 DPI, whereas their expression was greatly attenuated in *SlMS1* silenced fruit. Additionally, the expression of *PSY1*, *PDS* and *ZDS* was examined in *SlMS1* overexpressed fruit mediated by pSAK277-*SlMS1*. As shown in Figure 5D–F, *SlMS1* overexpression for 10 days induced an increased transcription of *PSY1, PDS* and *ZDS* significantly compared with the control fruit. For instance, the expression of *PSY1* in *SlMS1* overexpressed fruit was about 150 times that of the control. Therefore, *SlMS1* increased the accumulation of carotenoid possibly by increasing the transcription of carotenoid-synthesis-related genes *PSY1*, *PDS* and *ZDS*.

**Figure 6:**
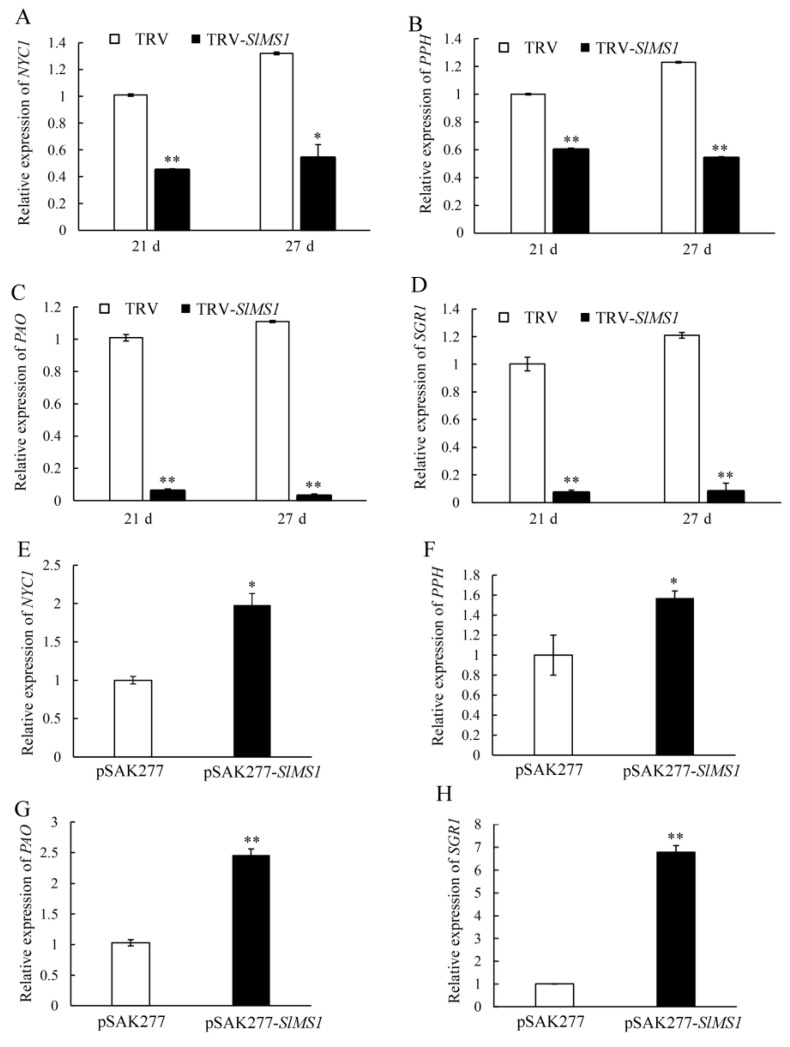
Effect of transient silencing and transient overexpression of *SlMS1* on gene expression of chlorophyll degradation related genes *NYC1*, *PPH*, *PAO* and SGR1 in tomato fruit. (**A**–**D**) Relative gene expression of *NYC1* (**A**), *PPH* (**B**), *PAO* (**C**) and *SGR1* (**D**) in control (TRV) and *SlMS1*-silenced fruit (TRV-*SlMS1*) at 21 and 27 days post inoculation. (**E**–**H**) Relative gene expression of *NYC1* (**E**), *PPH* (**F**), *PAO* (**G**) and *SGR1* (**H**) in control (pSAK277) and *SlMS1*-overexpressed (pSAK277-*SlMS1*) fruit at 10 days post inoculation. The symbols * and ** stand for *p* < 0.05 and *p* < 0.01, as determined by Student’s *t*-test, respectively.

Chlorophyll degradation is an important indicator of tomato fruit ripening. In order to explore the relations between *SlMS1* and the changes in chlorophyll content during the ripening process, we analyzed the key genes *NYC1*, *PPH*, *PAO* and *SGR1* in the chlorophyll degradation pathway. Whether it was at 21 DPI or 27 DPI, the contents of *PAO*, *PPH*, *NYC1* and *SGR1* in the gene-silencing fruit TRV-*SlMS1* were much lower than those in the control group, indicating that chlorophyll degradation may be slowed by decreased expression of chlorophyll degradation related genes compared to that of the control (Figure 6A–D). In contrast, the expressions of *PAO*, *PPH*, *NYC1* and *SGR1* in pSAK277-*SlMS1* were significantly higher than those in control fruit, of which *SGR1* was 6.2 times higher than that of control, indicating that the transient expression of *SlMS1* tomato fruit accelerated chlorophyll degradation by increasing the expression of chlorophyll degradation related genes (Figure 6E–H).

### 2.7. Effect of Gene Silencing and Overexpression of SlMS1 on the Expression of Ethylene Biosynthesis Genes

**Figure 7:**
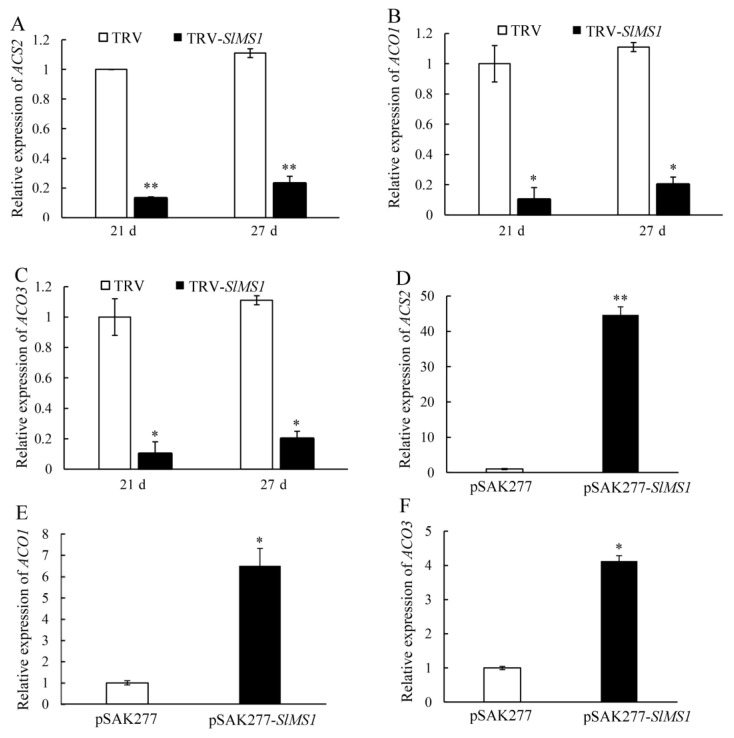
Effect of transient silencing and transient overexpression of *SlMS1* on gene expression of ethylene biosynthesis related genes *ACS2, ACO1* and *ACO3* in tomato fruit. (**A**–**C**) Relative gene expression of *ACS2* (**A**), *ACO1* (**B**) and *ACO3* (**C**) in control (TRV) and *SlMS1*-silenced fruit (TRV-*SlMS1*) at 21 and 27 days post inoculation. (**D**–**F**) Relative gene expression of *ACS2* (**D**), *ACO1* (**E**) and *ACO3* (**F**) in control (pSAK277) and *SlMS1*-overexpressed (pSAK277-*SlMS1*) fruit at 10 days post inoculation. The symbols * and ** stand for *p* < 0.05 and *p* < 0.01, as determined by Student’s *t*-test, respectively.

Ethylene is an important phytohormone in the ripening process of tomato fruit. In order to study the relationship between *SlMS1* and ethylene synthesis, the expression levels of ethylene-synthesis-related genes *ACS2, ACO1* and *ACO3* were determined in *SlMS1* silenced fruit and *SlMS1* overexpressed fruit. As shown in Figure 7A–C, the expression of *ACS2, ACO1* and *ACO3* increased gradually in both TRV-*SlMS1* and control fruit from 21 to 27 DPI, whereas TRV-*SlMS1* attenuated their expression greatly, whether at 21 DPI or 27 DPI, compared with control fruit; therefore, it could be concluded that *SlMS1* gene silencing resulted in a significant down-regulation of ethylene synthesis related genes. The expression level of *ACS2, ACO1* and *ACO3* in pSAK277-*SlMS1* at 10 DPI was much higher than that in the control group, among which *ACS2* was about 35 times higher than the control. These results indicate that *SlMS1* overexpression enhanced the transcription of genes involved in the ethylene synthesis pathway.

### 2.8. Effect of Gene Silencing and Overexpression of SlMS1 on the Expression of Cell-Wall-Metabolism-Related Genes in Tomato Fruit

**Figure 8:**
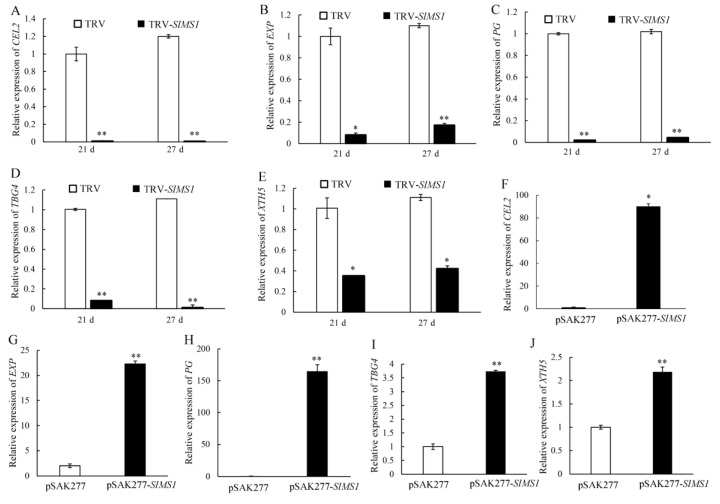
Effect of transient silencing and transient overexpression of SlMS1 on gene expression of cell wall metabolism related genes *CEL2, EXP, PG, TBG4* and *XTH5* in tomato fruit. (**A**–**E**) Relative gene expression of *CEL2* (**A**), *EXP* (**B**), *PG* (**C**), *TBG4* (**D**) and *XTH5* (**E**) in control (TRV) and *SlMS1*-silenced fruit (TRV-*SlMS1*) at 21 and 27 days post inoculation. (**F**–**J**) Relative gene expression of *CEL2* (**F**), *EXP* (**G**), *PG* (**H**), *TBG4* (**I**) and *XTH5* (**J**) in control (pSAK277) and *SlMS1*-overexpressed (pSAK277-*SlMS1*) fruit at 10 days post inoculation. The symbols * and ** stand for *p* < 0.05 and *p* < 0.01, as determined by Student’s *t*-test, respectively.

Tomato fruit is usually accompanied by a decrease in fruit firmness during the ripening process, and the softening of the pericarp is regulated by genes or enzymes related to cell wall metabolism. Therefore, the expression levels of genes *CEL2*, *EXP*, *PG*, *TBG4* and *XTH5* encoding cell wall metabolism related enzymes were analyzed in *SlMS1* silenced fruit and *SlMS1* overexpressed fruit. As shown in Figure 8A–E, compared with the control group, the expressions of *CEL2*, *EXP*, *PG*, *TBG4* and *XTH5* in TRV-*SlMS1* fruits were significantly down-regulated at both 21 and 27 DPI. Additionally, the expression levels of *CEL2*, *EXP*, *PG*, *TBG4* and *XTH5* in pSAK277-*SlMS1* fruits were significantly higher than those in the control group, and the expression levels of *CEL2* and *PG* were about 80 and 150 times higher than those in the control group (Figure 8F–J). In conclusion, the expression of genes related to fruit cell wall metabolism was significantly increased by the overexpression of *SlMS1*.

### 2.9. Correlation Analysis among Different Physiological Indices and Ripening-Related Gene Expression

**Figure 9:**
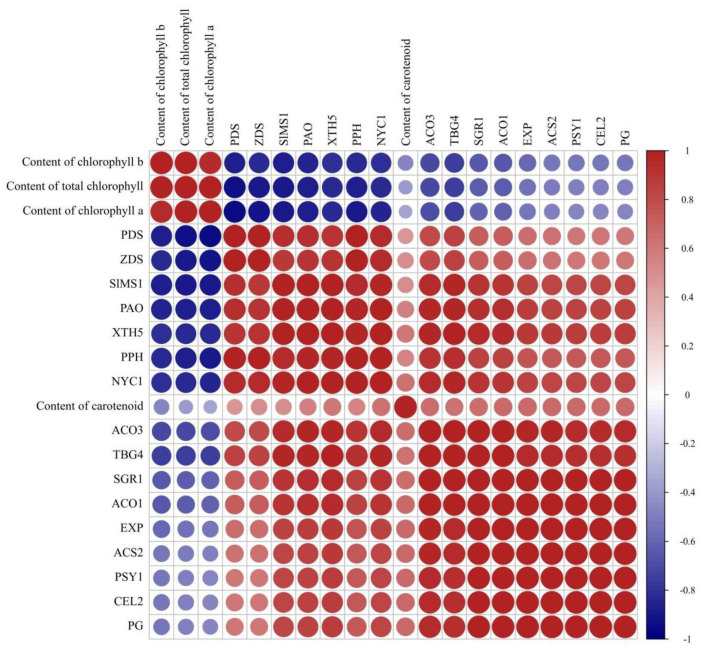
Correlation analysis of the parameters of chlorophyll, chlorophyll a, chlorophyll b, carotenoid, gene expressions of *NYC1, PAO, PPH, SGR1, PSY1, PDS, ZDS, ACS2, ACO1, ACO3, SlMS1, PG, XTH5, TBG4, EXP* and *CEL2* based on the data in control (TRV) and *SlMS1*-silenced fruit (TRV-*SlMS1*) at 21 and 27 days post inoculation and in control (pSAK277) and *SlMS1*-overexpressed (pSAK277-*SlMS1*) fruit at 10 days post inoculation.

To reveal the potential relation between *SlMS1* and fruit ripening, correlation was analyzed among *SlMS1*, the content of chlorophyll and carotenoids, and genes related to fruit ripening, including *NYC1*, *PAO*, *PPH*, *SGR1*, *PSY1*, *PDS*, *ZDS*, *ACS2*, *ACO1*, *ACO3*, *SlMS1*, *PG*, *XTH5*, *TBG4*, *EXP* and *CEL2*, based on the data determined. As shown in Figure 9, *SlMS1* is positively correlated with fruit-ripening-related genes and negatively correlated with chlorophyll, suggesting that *SlMS1* is indeed a positive regulator of fruit ripening. Moreover, total chlorophyll showed high negative correlation with the expression of chlorophyll degradation related genes *NYC1*, *PAO*, *PPH* and *SGR1*.

## 3. Discussion

The sulfur-containing amino acid methionine is essential for all organisms as a building block of proteins and as a precursor for the universal activated methyl donor SAM [19]. Ethylene biosynthesis starts with the conversion of SAM to ACC by ACS and then by ACO to ethylene [20]. Therefore, we propose that methionine content determines the influx to the ethylene pathway, and methionine synthase may participate in regulating fruit ripening. In the present work, the bioinformatics of methionine synthases in Arabidopsis and tomato were explored. All methionine synthases of Arabidopsis and tomato contained the Meth_synt_1 and Meth_synt_2 domains, and the amino acid sequence alignment and tertiary structure analysis suggested that they were highly conserved. Then, the expression patterns of *MS* genes in tomato were explored, and *SlMS1* showed an increasing pattern of expression during the early stage fruit ripening, suggesting that *SlMS1* might play an important role in regulating tomato fruit ripening. Previous research indicates that methionine synthase ER69, i.e., *SlMS1* in this work, is an ethylene-regulated gene, implying that methionine synthase may regulate tomato fruit ripening [14]. H_2_S is a negative regulator of fruit ripening and senescence in multiple postharvest fruits by antagonizing the effect of ethylene [15,16,17]. Thus, we proposed that *SlMS1* may be the target for the signaling role of H_2_S. By analyzing previously published transcriptome data [18], it was found that the expression of *SlMS1* in tomato fruit is greatly attenuated by H_2_S treatment, suggesting that H_2_S may delay fruit ripening by repressing the expression of *SlMS1*.

The first two reactions for de novo Met synthesis consist of the conversion of cysteine into homocysteine (Hcy) by the enzymes cystathionine γ-synthase and cystathionine β-lyase, and both enzymes are only present in the chloroplasts [12]. Plant methionine synthase seems to be present only in the cytosol [12,21,22], thus implying that Hcy has to exit the chloroplast to be converted into Met. Ravanel et al. [19] reported that AtMS1 and AtMS2 are cytosolic proteins, and the AtMS3-GFP fusion protein in protoplasts resulted in a pattern of green fluorescence, which colocalized with the red autofluorescence of chlorophyll. The potential localization of MS proteins is analyzed by an online tool, and the data in Table 1 suggest that SlMS1 shows cyto: 7, chlo: 3, mito: 2, nucl: 1, pero: 1, suggesting that SlMS1 primarily localizes in the cytoplast and, to a lesser extent, in the nucleus. Then, the detailed NLS of SlMS1 is predicted by NLS Mapper, and a bipartite NLS characterized by the basic amino acids KR and KK is found in the N-terminus of SlMS1, suggesting it may be a genius NLS. Consistent with the localization prediction, the fluorescence of recombinant *SlMS1*-GFP is observed mainly in the nucleus and partially in the cytoplasm in tobacco leaves. Therefore, we provide solid evidence that *SlMS1* shows both nucleus and cytoplasm localization, possibly due to the bipartite NLS. Accumulating evidence suggests that the active sulfur-related metabolism occurs in the cell nucleus. For instance, S-adenosylmethionine synthase MAT4, which is required for the modification of histone H3K9me2 and DNA methylation levels, also showed nuclear localization [23]. L-cysteine desulfhydrase SlLCD1, which is required for H_2_S, shows specific nuclear localization, and the nuclear localization is dependent on the NLS signal in its N-terminus [18]. The O-acetylserine(thiol)lyases SlOAS2, 4 and 6, were found to localize in the membrane, cytosol and nucleus, suggesting that cysteine could be synthesized in the nucleus [24]. A recent report indicates that methionine synthase METS1 in Arabidopsis acting as a nexus between the folate pathway and plant immunity and overexpression of METS1 represses plant immunity and is accompanied by a genome-wide global increase in DNA methylation [25], suggesting that methionine synthase is also involved in regulating plant immunity.

Methionine is a precursor for ethylene biosynthesis, and therefore, we propose that methionine synthase may participate in regulating fruit ripening. In the present work, we silence the expression of *SlMS1* in tomato fruit by VIGS and overexpress its expression by pSAK277 plasmid. The results indicate that the silencing of *SlMS1* delays tomato fruit ripening, while *SlMS1* overexpression promotes fruit ripening, implying that *SlMS1* is a positive regulator of fruit ripening. Moreover, the silencing of *SlMS1* reduces the accumulation of carotenoid and delays the degradation of chlorophyll. Then, the genes related to carotenoid biosynthesis and chlorophyll degradation are analyzed at the transcriptional level. The data indicate that *SlMS1* silencing caused a decreased expression of *PSY1*, *PDS* and *ZDS* compared with control during fruit ripening, whereas *SlMS1* overexpression for 10 days induces an increased transcription of *PSY1*, *PDS* and *ZDS* significantly. Further, *SlMS1* overexpression enhances the expression of *PAO*, *PPH*, *NYC1* and *SGR1*, while *SlMS1* silencing reduces their expressions. Thus, the work indicates that *SlMS1* overexpression in tomato fruit accelerates chlorophyll degradation and carotenoid biosynthesis by increasing the expression of chlorophyll degradation related genes and carotenoid biosynthesis related genes. Additionally, we find that *SlMS1* overexpression enhances the transcription of ethylene-synthesis-related genes, *ACS2*, *ACO1* and *ACO3*, and genes *CEL2, EXP, PG, TBG4* and *XTH5*, which encode cell wall metabolism related enzymes. To reveal the potential relation between *SlMS1* and fruit ripening, the correlation was analyzed among *SlMS1*, the content of chlorophyll and carotenoids and genes related to fruit ripening. The result suggests that *SlMS1* is positively correlated with fruit-ripening-related genes and negatively correlated with chlorophyll, suggesting that *SlMS1* acts as a positive regulator of fruit ripening.

**Figure 10:**
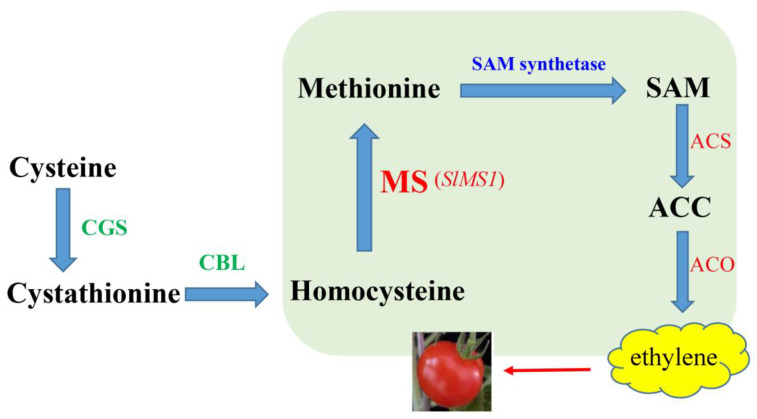
Metabolic diagram of methionine regulating fruit ripening. For methionine biosynthesis, CGS catalyzes the generation of cystathionine from cysteine and O-phosphohomoserine, and then, homocysteine is produced by CBL. The last step of Met synthesis is catalyzed by methionine synthase (MS encoded by *SlMS1*), which methylates homocysteine to form Met using N5-methyltetrahydrofolate as a methyl group donor. Methionine generates methyl donor SAM under the action of S-adenosylmethionine synthase, and then, ACC synthase (ACS) and ACC oxidase (ACO) catalyze the biosynthesis of ethylene. MS, methionine synthase; CBL, cystathionine β-lyase; CGS, cystathionine γ-synthase; SAM, S-adenosylmethionine; ACC, 1-aminocyclopropane-1-carboxylate.

Methionine synthase is responsible for methionine synthesis, and SAM accounts for about 80% of methionine metabolism, whereas the synthesis of proteins drives about 20% of methionine [11,19]. Thus, the expression of *SlMS1* is critical for initiating fruit ripening, as *SlMS1* may determine the content of methionine and also ethylene precursor, SAM. Finally, we propose a model of methionine and methionine synthase regulating fruit ripening, as shown in Figure 10. For methionine biosynthesis, CGS and CBL catalyze the pathway from cysteine to homocysteine via cystathionine. Then, methionine synthase (MS, encoded by ripening related gene *SlMS1*) methylates homocysteine to yield Met. Methionine generates methyl donor SAM under the action of SAM synthase, and then, ACC synthase (ACS) and ACC oxidase (ACO) catalyze the biosynthesis of ethylene. Therefore, *SlMS1* acts as a positive regulator of tomato fruit ripening, possibly through the synthesis of methionine, which leads to the influx into the ethylene biosynthesis pathway.

## 4. Materials and Methods

### 4.1. Identification, Phylogenetic Analysis and Data Exploration of Methionine Synthases in Plants

With the protein sequence of AtMS1 in Arabidopsis as a query, the homologous genes of methionine synthase in tomato and Arabidopsis were identified in https://phytozome-next.jgi.doe.gov/blast-search, accessed on 11 August 2022 by the program BLASTP-protein query to protein database. The protein molecular weights and theoretical pI values were computed using ProtParam (http://web.expasy.org/protparam/, accessed on 15 August 2022). The predicted NLSs were obtained from NLS mapper (http://nls-mapper.iab.keio.ac.jp/cgi-bin/NLS_Mapper_form.cgi, accessed on 12 August 2022) [26]. The subcellular localization of proteins was predicted on the website https://wolfpsort.hgc.jp/, accessed on 15 August 2022. The tertiary structure of proteins was predicted on the website of SWISS-MODEL (expasy.org).

The phylogenetic relationships among methionine synthases from *Arabidopsis thaliana* and tomato were investigated using MEGA 7.0 [27] with the neighbor-joining method and 1000 bootstrap replicates. Multiple sequence alignment was performed on the website https://espript.ibcp.fr/ESPript/ESPript/, accessed on 14 August 2022. The domain composition of proteins was predicted on SMART: Main page (embl-heidelberg.de). For the exploration of transcription of methionine synthase genes in different tissues of tomato, the original expression data were obtained from the online tool http://tomexpress.toulouse.inra.fr/query, accessed on 11 August 2022, and the tomato cultivar was Micro Tom.

### 4.2. Subcellular Localization Analysis

The CDS of *SlMS1* without a stop codon was inserted into the p1300-35S-GFP vector for *SlMS1*-GFP expression. *Agrobacterium tumefaciens* GV3101 containing the recombinant vectors was infiltrated into 5-week-old *N. benthamiana* leaves. Then, the tobacco was cultured at 22 °C under a 16 h light/8 h dark photoperiod for 2 days. The location of the nucleus was indicated by treatment with Hoechst 33342 (5 µg/mL) for 5 min. The excitation/emission wavelengths were 488 nm/507 nm for GFP and 346 nm/460 nm for Hoechst 33342. All fluorescence signals were detected using a Zeiss LSM710NLO confocal laser scanning microscope. The primers for the construction of *SlMS1*-GFP were shown in Table S2.

### 4.3. VIGS of SlMS1 and Transient Overexpression of SlMS1 in Tomato Fruit

A fragment of 400 bp corresponding to nt 1563–1964 of the *SlMS1* sequence was amplified from the cDNA of tomato by PCR and inserted into pTRV2 to yield pTRV2-*SlMS1* at restriction sites of EcoRI and BamHI. *Agrobacterium tumefaciens* GV3101 containing the vectors was cultured at 28 °C for 16 h in Luria-Bertani medium containing 20 μM AS, 10 mM MES and 50 μg/mL each of the antibiotics kanamycin, gentamycin and rifampicin, according to the method of Fu et al. [28]. Cells resuspended with the pTRV1 and pTRV2 or pTRV2-*SlMS1* vector were then mixed together at a ratio of 1:1 and infected into the pedicels of Micro Tom tomato plants. Tomato petioles infiltrated with pTRV2 without the insert were used as controls. Tomato plants were then stored at 16 °C for 24 h and transferred to normal culture conditions at 22 °C under a 16 h light/8 h dark photoperiod thereafter. The images of fruits were taken at 14, 21 and 27 days post injection. The samples without seeds were sampled at 21 and 27 days post injection and stored in a –80 °C refrigerator for subsequent measurement.

For transient overexpression of *SlMS1*, the coding sequence of *SlMS1* was cloned into the plasmid pSAK277 under the 35S promoter at the restriction sites of XbaI and EcoRI. *Agrobacterium tumefaciens* GV3101 containing pSAK277-*SlMS1* or the empty vector cultured overnight was diluted into 20 μM AS (Acetosyringone), 10 mM MES (2-Morpholinoethanesulphonic acid) to OD_600_ 0.8–1.0. After 4 h of culture, the Agrobacterium was injected into one chamber of the tomato fruit at the green mature stage. The infected plant was stored at 16 °C for 24 h in the dark and then placed in normal growth conditions. The images of fruits were taken at 5 and 10 days post injection. The samples without seeds were sampled at 10 days post injection and stored in a –80 °C refrigerator for subsequent measurement. The primers for the construction of pTRV2-*SlMS1* and pSAK277-SlMS1 were shown in Table S2.

### 4.4. Determination of the Levels of Chlorophyll and Carotenoids in Tomato Fruit

The tomato fruit sample at 0.5 g without the seeds was extracted in ethanol. Afterward, a quantitative determination of chlorophyll and carotenoids was carried out. The chlorophyll and carotenoids levels were measured and calculated based on the equations described by Lichtenthaler and Wellburn [29].

### 4.5. RNA Extraction and RT-qPCR

Reactions were conducted using previously reported methods [18]. Briefly, total RNA from 0.1 g of frozen samples was extracted using the RNA Extraction Kit (Tiangen, Beijing, China), and cDNA was synthesized from 500 ng total RNA in a reaction volume of 20 μL using a reverse transcription kit (PrimeScript RT Master Mix; Takara, Kyoto, Japan). The cDNA products were used for gene expression analysis by RT-qPCR. The RT-qPCR reaction system was 10 μL: 5 μL of 2 × SYBR^®^ Green Pro Taq HS Premix, 0.8 μL of cDNA, 0.2 μL of 10 μM F/R of each primer and 3.8 μL RNase free water. The reaction program was 95 °C for 30 s; 95 °C for 5 s; 60 °C for 30 s; 40 cycles, followed by melting curve at 95 °C for 15 s; 60 °C for 60 s; and 95 °C for 15 s. Using the tomato *Tubulin* gene as an internal reference, the relative expression was calculated according to the 2^−^^△△^^Ct^ method. Primers for the qRT-PCR analysis are listed in Table S3.

## Figures and Tables

**Table 1:** Nuclear localization prediction and physicochemical analysis of methionine synthases in *A. thaliana* and tomato.

Gene	Accession Number	Subcellular Localization Prediction	Number of Amino Acids	Molecular Weight	Theoretical pI
AtMS1	AT3G03780	cyto: 7, chlo: 4, mito: 2, nucl: 1	765	84583.84	6.09
AtMS2	AT5G17920	cyto: 5, mito: 4, chlo: 3, nucl: 1, pero: 1	765	84356.64	6.09
AtMS3	AT5G20980	mito: 9, chlo: 5	812	90594.12	8.17
SlMS1	Solyc10g081510	cyto: 7, chlo: 3, mito: 2, nucl: 1, pero: 1	765	84724.91	6.01
SlMS2	Solyc01g009180	mito: 9, chlo: 5	830	92074.57	6.74

## Data Availability

Data were based on three biological replicates in each experiment, and the experiments were repeated independently three times. The statistical analysis of the data was based on Student’s *t*-tests at *p* < 0.05.

## Supplementary Materials

The following supporting information can be downloaded at: https://www.mdpi.com/article/10.3390/ijms232012239/s1.
