# Supplementary material for: A Hydrogen-Sulfide-Repressed Methionine Synthase SlMS1 Acts as a Positive Regulator for Fruit Ripening in Tomato
Source: Int J Mol Sci. 2022 Oct 13;23(20):12239. doi: 10.3390/ijms232012239 (PMC9603753; doi:10.3390/ijms232012239)
Supplement: Supplementary file 1 [file ijms-23-12239-s001.zip › ijms-1915840-supplementary.pdf]

SUPPLEMENTARY MATERIAL

Supplementary Table S1. Transcription data of *SlMS1 and SlMS2* gene under 0, 1, 3 days of hydrogen sulfide (H_2S) treatment (0.6 mM NaHS as H_2S donor).

Gene	CK 0 d	H_2S 1 d	CK 1 d	H_2S 3 d	CK 3d
SlMS1	321.64	247.5	328.28	129.3	323.67
SlMS2	21.68	20.37	17.06	14.88	20.85

Supplementary Table S2. Primers for VIGS-*SlMS1 and SlMS1*-pSAK277 vectors.

Gene	Primers	Sequences	Amplicon size (bp)
Solyc10g081510.1.1	*SlMS1*-277-F	ACTAGTGGATCCAAAGAATTCATGGC ATCTCATGTTGTTGGATAT	2298
	SlMS1-277-R	TCATTAAAGCAGGACTCTAGATCACTT GGCGCTGGCGAG	
	VIGS-*SlMS1*-F	GTGAGTAAGGTTACCGAATTCATGTG TGAAGCCACCAATCATCT	400
	VIGS-*SlMS1*-R	CGTGAGCTCGGTACCGGATCCGTTGA AGTTGGAGTAGCACATGTGA	
	GFP-*SlMS1*-F	GAGAACACGGGGGACTCTAGAATGG CATCTCATGTTGTTGG	2298
	GFP-*SlMS1*-R	GCCCTTGCTCACCATGGATCCCTTGGC GCTGGCGAGC	

Supplementary Table S3. Primers used for quantitative RT-PCR.

Primers	Sequence	Gene ID	Amplicon size (bp)
SlSGR1 (forward)	GGCTATCTCCCAAACCATCAA	Solyc08g080090	80
SlSGR1 (reverse)	ACTCTGCAACAACTTCATCTCT		
SlPPH (forward)	CAGATGTCGTGGATGGGAAA	Solyc01g088090	101
SlPPH (reverse)	GCCTTTAGCTTCACTTGGTAAAC		
SlPAO (forward)	CCTCATCGTCTTGCTCCTTTAT	Solyc11g066440	119
SlPAO (reverse)	GCAGCTTGAGGTATCCTTGT		
SlNYC1 (forward)	GATCAGGGAAGAGCACTGTATG	Solyc07g024000	128
SlNYC1 (reverse)	GAGAACACAGACACCCAAGTAT		
SlPSY1 (forward)	GAAGATGCCAGAAGAGGAAGAG	Solyc03g031860	93
SlPSY1 (reverse)	GGTCACCCTTCCAGCAAATA		
SlPDS (forward)	CAAGACCAGAGCTGGACAATAC	Solyc03g123760	119
SlPDS(reverse)	CAAACCTGCACCAGCAATAAC		
SlZDS (forward)	GATTGGTTCCTCAGAAGTGGAG	Solyc01g097810	101
SlZDS (reverse)	CCAGCCATTGTAGCGTAGTT		
SlACO1 (forward)	CCATGTCCTAAGCCCGATTT	NM_001247095.2	94
SlACO1 (reverse)	GGCCACTCACTTTGTCATCT		
SlACO3 (forward)	GCGCCATCTTCCTACTTCTAAT	Solyc07g049550	103
SlACO3 (reverse)	CTCAGCCAACTTCTCCAATCT		

SlACS2 (forward)	GAGGTTCGTAGGTGTTGAGAAA	Solyc01g095080	147
SlACS2 (reverse)	GGAGGAATAGGTGACGAAAGTG		
SlPG (forward)	TTGGAGGAGGAGGAACTATCA	Solyc10g080210	83
SlPG (reverse)	CCTGCATGGCAGTGATTTATTT		
SlCEL2 (forward)	CTGCTGTTTGCCCTTTCTATTG	Solyc09g010210	94
SlCEL2 (reverse)	TGCCTTCTTCTTGTTGCTTTATG		
SlEXP (forward)	CCGACGATTGGACACCTAAA	Solyc06g051800	105
SlEXP (reverse)	GGTCCTCCTTAATCAAAGGACATA		
SlTBG4 (forward)	GCCATTGGCCTGGATACATA	Solyc12g008840	101
SlTBG4 (reverse)	AGAAGGTTGTCCGCAGTTAG		
SlXTH5 (forward)	AGGATTCAGCCATCTCTTTGG	Solyc01g081060	101
SlXTH5 (reverse)	ACTTGAACCCTGAACCTGTG		
SlTubulin (forward)	TAGAGCCTGGTACGATGGATAG	Solyc08g006890	134
SlTubulin (reverse)	CAACTCAGCGCCTTCAGTATAA		

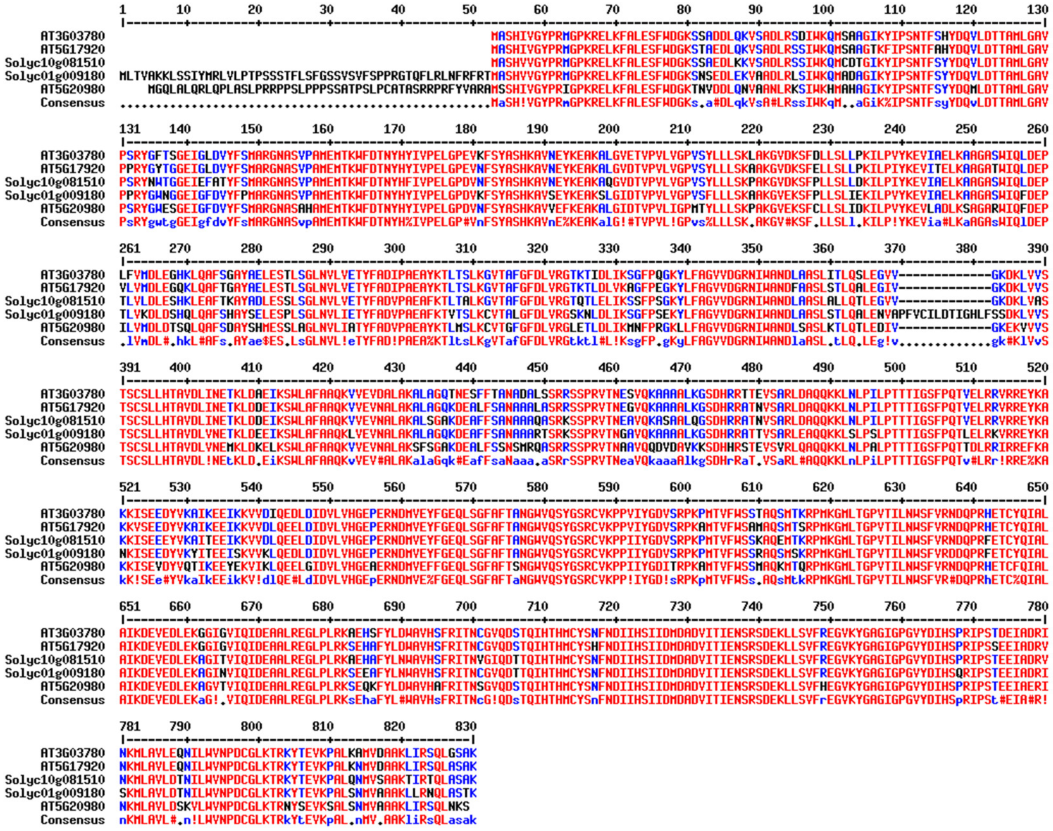

Supplementary Figure S1. Amino acid sequence alignment of methionine synthases in Arabidopsis and tomato.

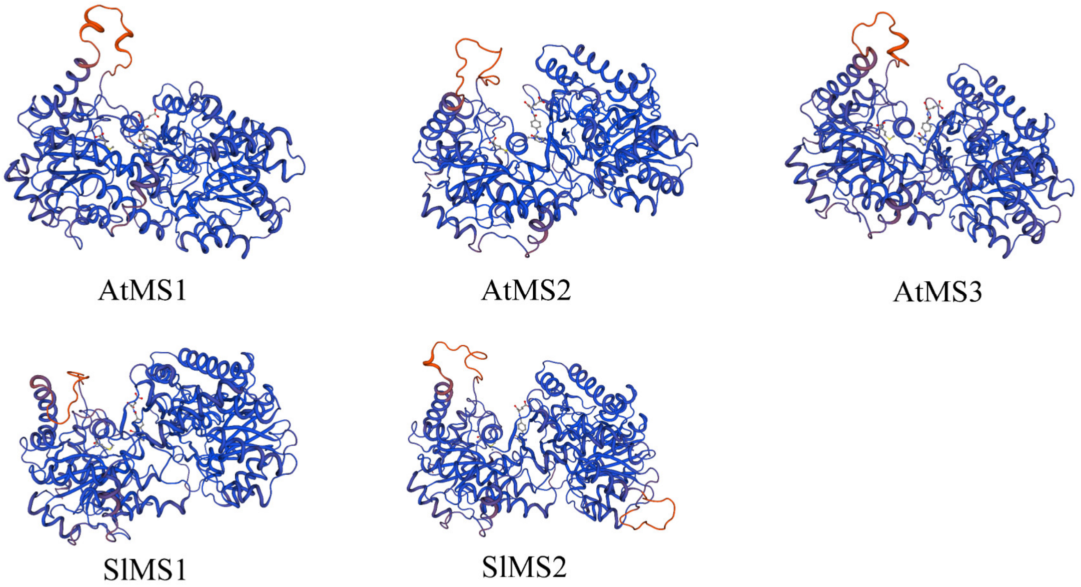

Supplementary Figure S2. Tertiary structures of methionine synthases in Arabidopsis and tomato.
